# Above-ground carbon stock and REDD+ opportunities of community-managed forests in northern Thailand

**DOI:** 10.1371/journal.pone.0256005

**Published:** 2021-08-18

**Authors:** Siriluck Thammanu, Hee Han, Dokrak Marod, Jamroon Srichaichana, Joosang Chung

**Affiliations:** 1 Royal Forest Department, Bangkok, Thailand; 2 Department of Forest Policy and Economics, National Institute of Forest Science, Seoul, Korea; 3 Department of Forest Biology, Faculty of Forestry, Kasetsart University, Bangkok, Thailand; 4 Geography Department, Thaksin University, Songkhla, Thailand; 5 Department of Agriculture, Forestry and Bioresources, Seoul National University, Seoul, Korea; 6 Research Institute of Agriculture and Life Sciences, Seoul National University, Seoul, Korea; Feroze Gandhi Degree College, INDIA

## Abstract

This study aimed to investigate the structure of two deciduous forests and assess their above-ground carbon stock in order to promote community forest management (CFM) for REDD+ opportunities in the Ban Mae Chiang Rai Lum Community Forest in northern Thailand. A systematic sampling method was used to establish twenty-five sample plots of 40 m × 40 m (0.16 ha) each that were used to survey the entire 3,925 ha area of the community forest. Cluster analysis identified two different forest types: dry dipterocarp forest and mixed deciduous forest. It was determined that the above-ground carbon stock did not vary significantly between them. An analysis of carbon sequestration in the community forest indicates that carbon stock increased under CFM from 2007 to 2018 by an estimated 28,928 t C and participation in the carbon market would have yielded approximately US $339,730.43 or US $8.66 /ha/year to the community for that 10-year period. Projections for 2028 reflect that carbon stock will experience continual growth which indicates that maintaining CFM can increase carbon sequestration and reduce CO_2_ emissions. However, though further growth of carbon stock in the community forest is expected into 2038, that growth would be at a lesser rate than during the preceding decade. This suggests that CFM management should address forest utilization practices with a focus on maintaining long term carbon stock growth. Additional measures to address the impact of drought conditions and to safeguard against forest fires are required to sustain tree species’ growth and expansion in order to increase their carbon accumulation potential. Thailand’s community forest involvement in REDD+ and participation in its international carbon market could create more economic opportunities for local communities.

## Introduction

Forests provide essential environmental, social and economic benefits to local communities. They are one of the world’s largest carbon sinks storing 45% of the earth’s terrestrial carbon and absorbing 2.4 billion tons of atmospheric CO_2_ each year [1–3]. This carbon can result in economic and other benefits to communities, such as through participation in the Reducing Emission from Deforestation and Forest Degradation (REDD+) program’s international carbon market [4, 5], as Thailand and many other countries are doing [6].

Vast amounts of carbon can be found in above-ground biomass in amounts that vary based upon forest type, tree species composition, diversity, and other factors [7–9]. Understanding these factors and having accurate sequestration data can inform the efficacy of pursuing the myriad benefits of participating in international carbon markets [10].

Thailand’s forest resources are among the most abundant in Southeast Asia. Forests cover 31.68% of the country’s area of which 18.26% is deciduous forests [11]. These deciduous forests consist primarily of dry dipterocarp (DDF) and mixed deciduous (MDF) forests [12–15].

A deciduous forest is composed of numerous tree species that reflect local climates, topography, and soil conditions [14]. A DDF will have a mean annual rainfall of 1,000–1,500 mm compared with 1,000–1,800 mm in MDFs [16, 17]. DDFs can be found below 500 m elevation, while MDFs are found at elevation up to 900 m [13, 14, 18]. The soil upon which an MDF will grow is typically moderate fertile loam soil while DDF soil is more sandy and lateritic [16, 17, 19]. Deciduous forests regularly feature forest fires and lengthy drought periods of 5–6 months [18, 20]. The dominant tree species generally found in a DDF are *Dipterocarpus* spp., *Shorea obtusa*, *S*. *siamensis*, *Sindora siamensis*, *Xylia xylocarpa*, *Pterocarpus macrocarpus*, and *Irvingia malayana*. The MDF is composed of very distinctive dominant species such as *Tectona grandis*, *Shorea siamensis*, *Dillenia pariflora*, *Pterocarpus macrocarpus*, *Xylia xylocarp*, *Afzelia xylocarpa*, and *Lagerstroemia calyculatus* [14, 18, 21, 22].

In Thailand’s northern region, MDFs are the primary forest type and provide valuable timber; DDFs are good sources of income-supplementing non-timber forest products in remote areas [14, 23–25]. As such, deciduous forests are more useful for rural communities as a source of forest products and as high capacity carbon sinks [26, 27].

Previous studies have demonstrated that local forest resource management can enhance carbon sequestration and reduce the CO_2_ emissions that would otherwise be caused by deforestation and forest degradation [28–32]. Community forest management (CFM) has generally been accepted as a principle for sustainable management through collaboration between local people and governments [33]. In Thailand, CFM has been promoted by the Royal Forest Department (RFD) since 1987 [34]. Most recently, the Thai government approved Community Forest Act B.E 2562 that authorized local management decision-making [35].

In the Paris Agreement of the 2015 United Nations Climate Change Conference (COP21), Thailand announced its intention to adopt a low-carbon growth path and reduce its emissions by 20% by year 2030 [6]. Locally managed community forest projects can have a positive impact on carbon storage and sequestration and ultimately on the gas emissions caused by deforestation and forest degradation. During more than three decades of CFM’s prevalence, 1.2 million ha, or 7% of Thailand’s total forest area in 17,400 villages have come under CFM [36] demonstrating a nationwide proliferation of local community empowerment in managing forest resources. A more nationwide approach through implementation of the REDD+ policies and strategies and involvement in the international carbon market can magnify the positive impact while addressing the inherent challenges of attaining the country’s goal of reducing gashouse gas emissions.

However, there is a lack of information about the carbon sequestration potential of community forests in Thailand’s deciduous forests and how such data can be used to thoroughly understand the ecological and economic impact of sustainable forest management.

Our study would be a good model to demonstrate to other communities that the more expansive, global conservation policies, strategies and the carbon market mechanism of REDD+ can offer significantly more protection to the forest as well as bring enhanced economic benefit. Being the case, the objectives of this study were to 1) identify the structural differences in the deciduous forest types, 2) estimate above-ground biomass and carbon stock in a community forest, and 3) evaluate the impact of CFM on reducing the carbon emissions caused by deforestation and forest degradation.

## Materials and methods

### Study site

**Fig 1:**
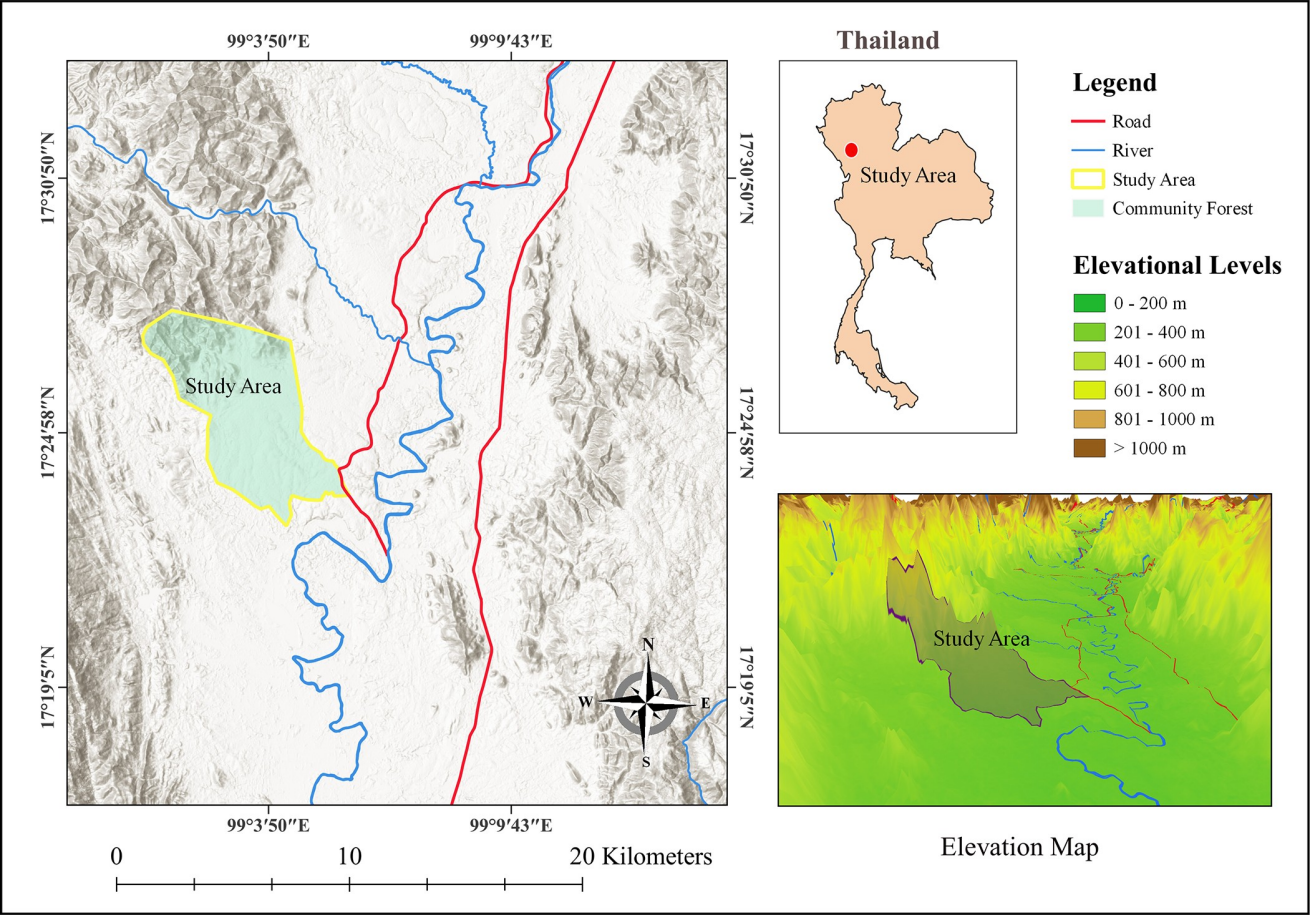
Location of the Ban Mae Chiang Rai Lum Community Forest in northern Thailand.

Ban Mae Chiang Rai Lum Community Forest of the Pa Mae Phrik National Forest Reserve was chosen as the study area. It is in the northern Thailand province of Lampang (N 17° 22’ 48" to N 17° 27’ 47" and E 99° 00’ 47" to E 99° 05’ 48") (Fig 1), has an area of 3,925 ha, and an elevational range of 140–660 m. Two deciduous forest types, dry dipterocarp and mixed deciduous, were identified in this community forest. The study site was located in a National Forest Reserve area under the exclusive authority of the Royal Forest Department of Thailand. As the study was conducted by staff of the Royal Forest Department, no access permits were required.

The study area features two distinct seasons, a wet season from April to October and a dry season from November to March. During the dry season, regular and prolonged drought conditions are experienced. Average temperatures ranged from 31.7°C in January and November to 37.1°C in March. Mean annual rainfall was 1,129.4 mm, the mean annual temperature was 33.6°C and mean relative humidity was 76.1% [37].

Since 2008, local people in collaboration with the RFD have managed the Ban Mae Chiang Rai Lum Community Forest under a community forest project. Previous widespread damage caused to the land by encroachment and illegal logging and the impact on livelihoods prompted this action. Management policies were implemented to safeguard the forest and its benefits. Surveys, alignment and patrols, preservation of cultural and traditional activities, passing community regulations addressing the utilization of resources, and taking fire protection measures were all part of the overall management effort.

The distinct ecological characteristic of the two forest types informed different strategies for each. Plantation and restoration projects were established in the MDF. Check dams (cross-stream structures created using natural materials such as rock, branches, or sandbags to slow water flow, control erosion and increase hydration) were constructed in the DDF to promote and foster tree species diversity and to address the need for an ongoing supply of NTFPs. Moreover, acquiring and transferring the skill and knowledge required to manage the forest appropriately while raising public awareness of the importance and value of the forest are all crucial components to conservation and sustainable management of the community forest.

Under CFM, less frequent forest fires resulted in greater soil moisture and increased water supplies making the community forest more conducive to growth and regeneration. Higher income and improved livelihoods were realized concomitant with a more widespread appreciation of the importance and value of a healthy forest abundant with NTFPs [38]. This is consistent with previous studies that found conserving forest resources enhances the economic benefits provided to local communities [39–43].

### Data collection

From July to October 2018, a sampling survey was conducted. The sampling intensity and precision were calculated using the results of a 2016 study by the RFD in the same area [44]. Confidence probability was 95%. The standard deviation from previous surveys was used to obtain an estimate of the sampling [45]. In a natural forest such as the study area, there would be greater variation, and an accuracy required estimate of 20% would be satisfactory [46]. The formula was expressed as:

$$n={\left(Z\sigma /E\right)}^{2}$$ (1)

where n is the sampling intensity; Z is the z-value for the confidence interval of 95%; *σ* is the density of tree species, and E is the percentage of standard deviation from the required precision.

**Fig 2:**
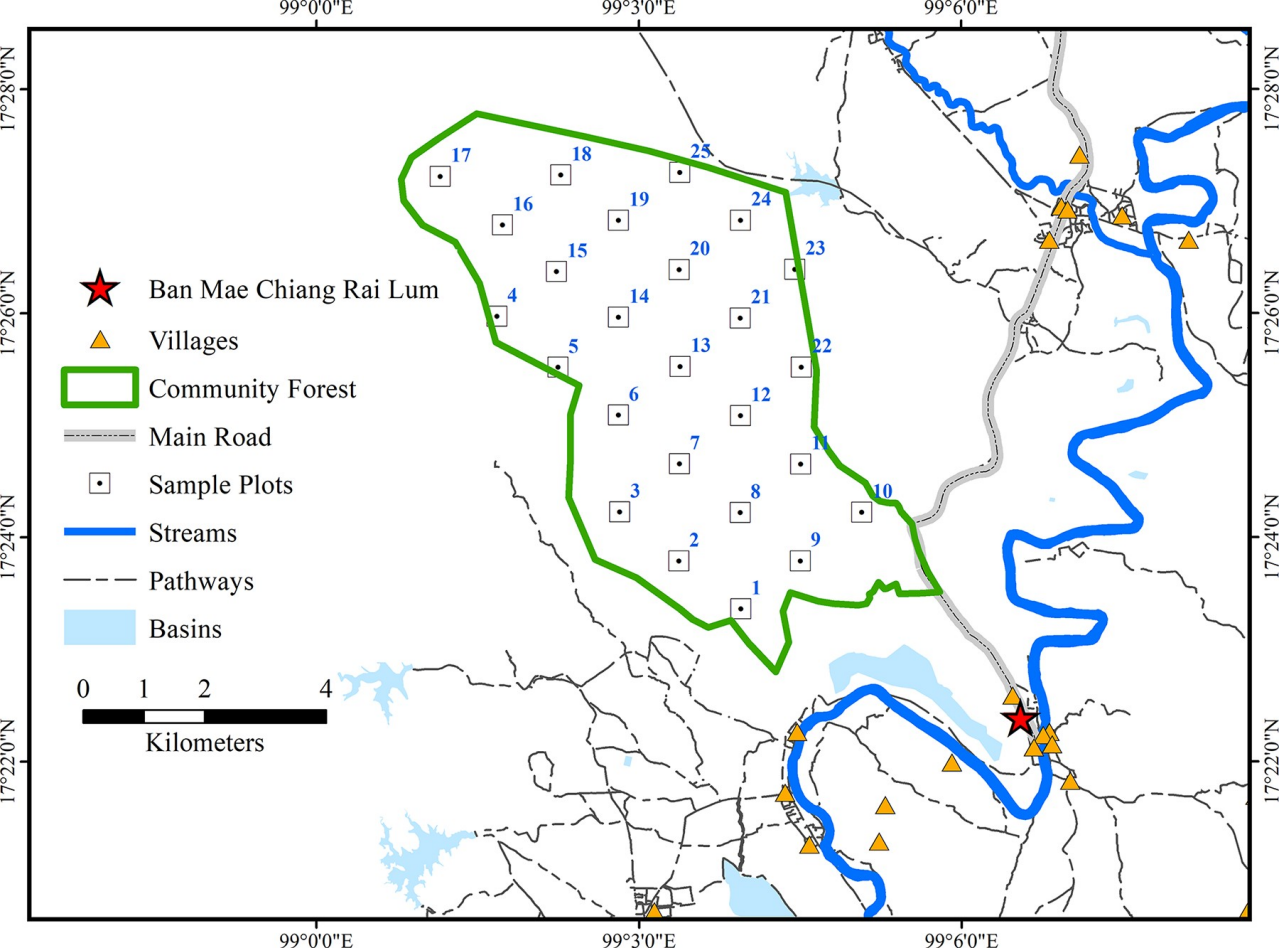
Location of the sampling plots identified through the systematic sampling method.

A systematic sampling method was used to survey 25 sampling plots measuring 40 m × 40 m (0.16 ha) each which were separated by an average of roughly 1,200 m [47, 48]. Each 40 m × 40 m plot was divided into 16 distinct 10 m × 10 m subplots to measure and identify trees with a diameter at breast height (DBH) ≥ 5 cm [49]. The locational information of the sample plots is shown in Fig 2.

### Data analysis

The two distinct forest types in the community forest were identified by cluster analysis employing importance value index (IVI) matrices in each sampling plot. A cluster analysis requires pruning of the dendrogram at a level representing a compromise between the group and the number of groups. Optimum pruning for the dendrogram was selected by applying the Euclidean distance [50].

Trees (DBH ≥ 5 cm) were identified into family and species to determine the ecological characteristics of the forest types. Identification of some species was only accomplished through a comparison with samples at the Forest Herbarium, Department of National Parks, Wildlife and Plant Conservation Herbarium. The density and basal area of each tree species were calculated and the diversity of the trees in the community forest was examined by analyzing the Shannon-Wiener index (H′) [51]. The applied equation was:

$$H^{\prime }=-\sum_{i=1}^{s}\left(pi)({log}_{2}pi\right)$$ (2)

where s is number of species; pi is the proportion of individuals found in the i^th^ species.

To compare the species composition of the two forest types, the Jaccard similarity index was calculated [52] and the differences in the species, density, basal area, and diversity index of the two forests were subjected to a one-way analysis of variance (ANOVA).

In addition, the IVI using the following equation was applied to quantify the ecological importance of the tree species in each forest:

$$IVI=R.D+R.F+R.D_{o}$$ (3)

where R.D is the relative density of the tree species; R.F is their relative frequency, and R.D_o_ is their relative dominance. They were calculated as R.D = number of individuals of the species × 100 / total number of quadrate studies, R.F = number of quadrates in which species occurred × 100 / total number of quadrate studies, and R.D_o_ = total basal area of species × 100 / total basal area of all the species [53].

To calculate tree biomass, we used allometric equations following Ogawa et al. (1965) for natural deciduous forests in Thailand (dry dipterocarp and mixed deciduous forests) to compute above-ground biomass including stems, branches, and foliage [54]. The following allometric equations were used:

$$\begin{array}{l} Above‐Ground\;Biomass=Ws+Wb+Wl\\ Ws=0.0396{\left(D^{2}H\right)}^{0.9326}\\ Wb=0.003487{\left(D^{2}H\right)}^{1.027}\\ Wl={\left((28.0/Ws+Wb)+0.025\right)}^{‐1} \end{array}$$ (4)

where D = diameter at breast height (cm), H = height of tree (m), Ws = stem biomass (t ha^-1)^, Wb = branch mass (t ha^-1^), and Wl = leaf mass (t ha^-1^).

To estimate the carbon stock, we converted above-ground biomass into carbon stock using the IPCC default 0.47 carbon fraction [10], and the estimates of the carbon stock in the two deciduous forests were compared. The formula for this calculation is:

Carbonstock=Biomass×0.47 (5)

In addition, to investigate the impact of CFM on the reduction of carbon emissions caused by forest deforestation and degradation, a Random Forest (RF) classifier was used to classify the community forest. Landsat data was improved and used in the mapping of forest degradation by mixing field surveys, Normalized Difference Vegetation Index (NDVI), and Digital Elevation Model (DEM) data [55]. The CA-Markov method was implemented for a Land Use and Land Cover (LULC) prediction of future restoration of the community forest.

The satellite data used in this study were Landsat-5 Thematic Mapper (TM) and Landsat-8 Operational Land Imager (OLI) images. The Landsat images were downloaded from the United States Geological Survey (USGS) as standard Level-2 topographic with corrected surface reflectance. The multi-spectral image data were then improved by DEM data derived from Shuttle Radar Topography Mission (SRTM) and scene number SRTM1N17E099V3. The overview of our image data is shown in Table 1.

**Table 1:** Basic information of Landsat data and SRTM DEM.

Satellite	Date of acquisition	Path/Row	Bands with SRTM DEM
**Landsat-5**	22 February 2007	130/48	1–5, 7, and SRTM DEM
**Landsat-8**	11 February 2018	131/48	2–7 and SRTM DEM

An RF classifier was applied to all Landsat images and SRTM DEM from 2007 and 2018 resulting in the classification of the community forest into two forest types: dry dipterocarp and mixed deciduous forests [56]. The overall accuracy and kappa statistics were expanded for an accuracy assessment over 80%. An interpretation by Erdas software with a change detection function informed three distinct areas of the forest: degraded, restored, and retained. We used the CA-Markov model to simulate community forest changes and predict variations with images. The CA-Markov model was used to predict restorative changes in the community forest for 2028 and 2038 based on changes reflected in satellite images in the 2007 to 2018 maps, and its validity was investigated [57]. In addition, the CA-Markov model was developed from transition probability matrices in the community forest representing the changes for 2007 and 2018. The transition probability matrices for 2028 and 2038 were derived from the CA-Markov model; the spatial data reflected predicted changes in the community forest and estimated above-ground biomass and carbon stock [9, 58, 59].

All the statistical calculations were performed using version 5.10 of PC.ORD [60] and version 3.6.2 (2019-12-12) of the R program for Windows software [61]. ArcMap version 10.5 was applied to Landsat images to analyze the Random Forest classifier. The CA-Markov method in IDRISI software was applied to project the changes in the community forest.

## Results

### Forest classification

**Fig 3:**
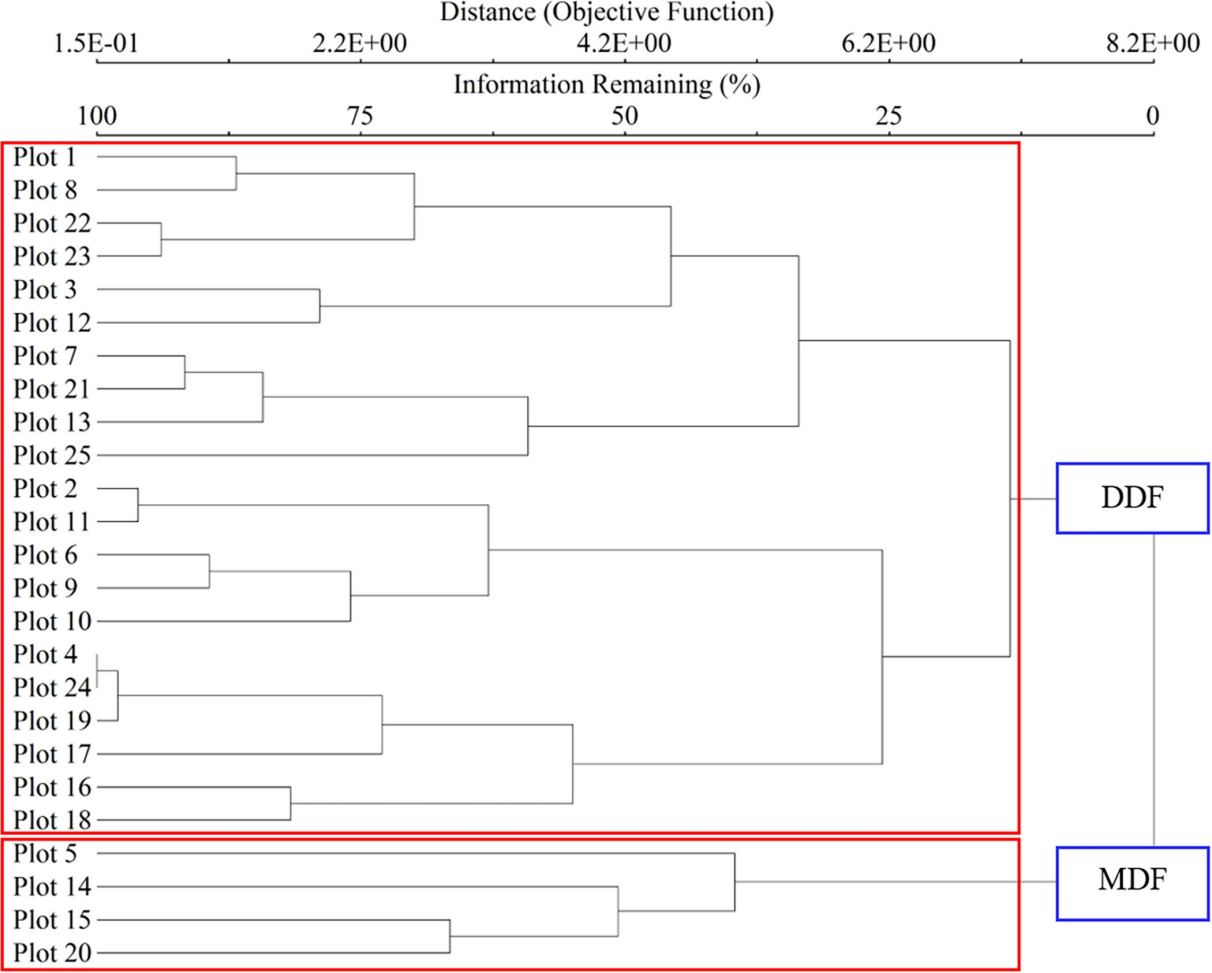
Classification of the forests in the deciduous area of the Ban Mae Chiang Rai Lum Community Forest in northern Thailand: Dry dipterocarp forest (DDF) and mixed deciduous forest (MDF).

The resulting dendrogram reflected 5.83% chaining and was cut with 12.5% of the remaining information explained by two forest types: dry dipterocarp forest (DDF) and mixed deciduous forest (MDF). The DDF contained 21 plots: plots 1, 2, 3, 4, 6, 7, 8, 9, 10, 11, 12, 13, 16, 17, 18, 19, 21, 22, 23, 24, and 25. The MDF contained 4 plots: plots 5, 14, 15, and 20 (Fig 3).

### Forest structure, species composition and diversity

The inventory of the Ban Mae Chiang Rai Lum Community forest yielded a total of 3,769 trees covering 129 species and 43 plant families. There were 2,992 trees in 93 species belonging to 36 families in the DDF. The highest numbers of species were from the following families: Rubiaceae, Euphorbiaceae, Papilionoideae, Anacardiaceae, and Caesalpinioideae. The MDF was populated by 777 trees of 72 species in 31 families. The highest numbers of species were found in the Papilionoideae, Rubiaceae, Caesalpinioideae, Euphorbiaceae, and Lythraceae families. The Jaccard index revealed a 28.68% similarity as 37 of the 129 total species were found in both the DDF and the MDF. The ANOVA analysis reveals that there was significant difference in species (F = 6.435; p < 0.05). However, the differences between the two forests in density (F = 0.155; p > 0.05), basal area (F = 4.069; p > 0.05), and Shannon-Wiener index (H′) (F = 2.677; p > 0.05) were not statistically significant. The ecological characteristics in each forest are shown below in Table 2.

**Table 2:** Ecological characteristics of deciduous forests (Mean ± Standard deviation) in the Ban Mae Chiang Rai Lum Community Forest, Northern Thailand.

Characteristics	DDF	MDF	Average
**Number of species** *	22.57 ± 5.46	31.00 ± 9.27	23.92 ± 6.74
**Density (tree ha** ^**-1**^ **) N.S.**	890.48 ± 220.60	1,214.06 ± 581.74	942.25 ± 312.27
**Basal area (m** ^**2**^ **ha** ^**-1**^ **) N.S.**	16.29 ± 3.97	17.16 ± 4.45	16.43 ± 3.96
**Shannon-Wiener index (H′) N.S.**	2.44 ± 0.28	2.68 ± 0.16	2.48 ± 0.28

N.S. = not significant at

*p < 0.05. DDF = dry dipterocarp forest, MDF = mixed deciduous forest.

**Fig 4:**
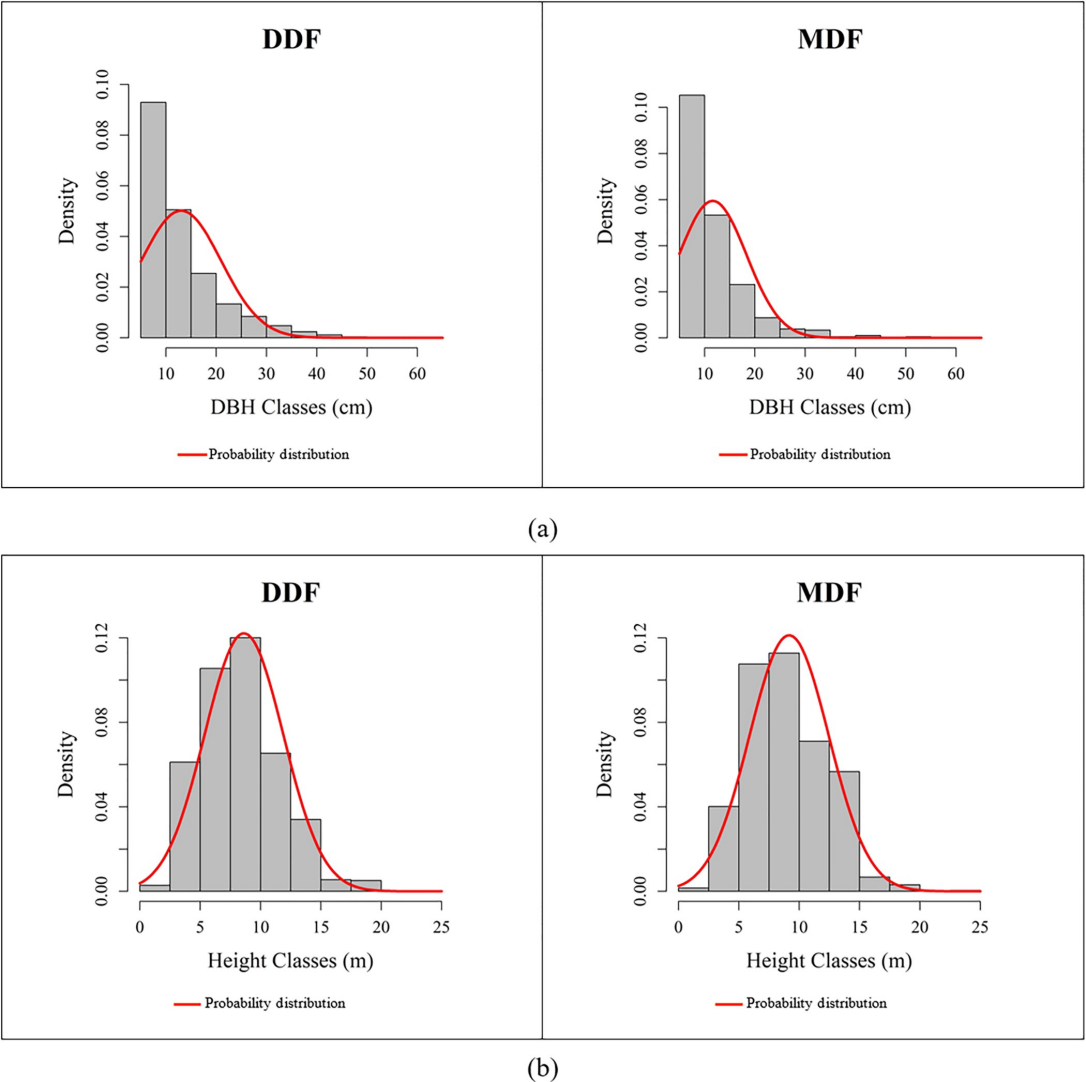
Distribution of the 3,769 trees in (a) DBH-class and (b) height-class within the deciduous forests.

The tree density distribution of diameter and height classes of the deciduous forests is shown in Fig 4. The DDF had a DBH range of 5.00–64.34 cm with a mean of 13.03 ± 7.95 cm. More specifically, trees with DBH < 10 cm (40.46%) were the most abundant followed by trees with DBH 10–20 cm (39.97%), 20–30 cm (10.89%), and > 30 cm (4.68%). In the MDF, DBH varied between 5.00–53.80 cm with a mean of 11.62 ± 6.71 cm. Specifically, trees with DBH < 10 cm (52.64%) were the most abundant followed by trees with DBH 10–20 cm (38.22%), 20–30 cm (6.31%), and > 30 cm (2.83%).

The height of the trees in the DDF ranged between 1.30–25.00 m with a mean height of 8.62 ± 3.27 m. The height of the trees in the MDF varied between 2.00–25.00 m with a mean height of 9.16 ± 3.29 m. In the DDF and MDF, respectively the 5–10 m class (56.38%, 55.08%) had the highest density followed in descending order by the 10–15 m class (24.83%, 31.92%), 1.3–5 m class (15.98%, 10.42%), and the > 15 m class (2.81%, 2.57%). Overall, the DBH ranged from 5 to 64.34 cm with a mean of 12.73 ± 7.73 cm. Height ranged from 1.30 m to 25 m with a mean of 8.73 ± 3.28 m.

As reflected in Table 3, the ten species with the highest IVI in the deciduous forests belong to seven families: Dipterocarpaceae, Mimosoideae, Caesalpinioideae, Burseraceae, Papilionoideae, Lythraceae, and Euphorbiaceae. The most dominant tree species in the DDF were *Shorea obtusa* (15.18%), *S*. *siamensis* (11.63%), *Xylia xylocarpa* (8.20%), *Sindora siamensis* (6.63%), and *Canarium subulatum* (4.85%). In the MDF, *Millettia leucantha* (8.48%) had the highest IVI, followed in decreasing order by *Lagerstroemia duperreana* (7.30%), *Millettia brandisiana* (7.18%), *Antidesma sootepense* (6.06%), and *Pterocarpus macrocarpus* (4.88%).

**Table 3:** Importance value index (IVI) of the five most important species in each forest.

Forest types/Ranking		Species	Relative values (%)			
			R.D	R.F	R.D_o_	IVI
**DDF**	1)	*Shorea obtusa*	4.58	3.99	6.62	15.18
	2)	*Shorea siamensis*	4.23	2.81	4.58	11.63
	3)	*Xylia xylocarpa*	2.89	3.26	2.06	8.20
	4)	*Sindora siamensis*	2.76	1.34	2.53	6.63
	5)	*Canarium subulatum*	0.90	1.26	2.69	4.85
		88 other species	17.97	20.67	14.86	53.50
**MDF**	1)	*Millettia leucantha*	3.86	2.11	2.51	8.48
	2)	*Lagerstroemia duperreana*	2.45	2.11	2.75	7.30
	3)	*Millettia brandisiana*	2.62	2.02	2.54	7.18
	4)	*Antidesma sootepense*	3.30	1.93	0.82	6.06
	5)	*Pterocarpus macrocarpus*	0.94	1.40	2.53	4.88
		67 other species	20.16	23.77	22.18	66.11

DDF = dry dipterocarp forest, MDF = mixed deciduous forest. R.D = relative density, R.F = relative frequency, R.D_o =_ relative dominance, IVI = importance value index.

### Above-ground biomass and carbon stock

The estimates of above-ground biomass and carbon stock are illustrated in Table 4. The total above-ground biomass of DDF was 63.60 ± 27.80 t ha^-1^: stem 51.43 ± 19.79 t ha^-1^, branch 10.34 ± 4.40 t ha^-1^, and foliage 1.82 ± 0.62 t ha^-1^. The MDF total above-ground biomass was estimated at 69.67 ± 21.83 t ha^-1^: stem 56.54 ± 17.60 t ha^-1^, branch 11.07 ± 3.67 t ha^-1^, and foliage 2.06 ± 0.60 t ha^-1^. The average above-ground biomass throughout the community forest was 64.57 ± 24.02 t ha^-1^. Analyzing the conversion of biomass to carbon stock, we find that the MDF provided higher volumes of carbon stock (32.74 ± 10.26 t C ha^-1^) than did the DDF (29.89 ± 11.65 t C ha^-1^). The ANOVA test showed that the differences in carbon stock between the two forests did not vary significantly (F = 0.280; p > 0.05). The average carbon stock in the entire community forest was 30.35 ± 11.29 t C ha^-1^.

**Table 4:** Estimation of biomass and carbon stock (Mean ± Standard deviation) in Ban Mae Chiang Rai Lum Community Forest.

Forest types	Above-ground biomass (t ha^-1^)				Carbon stock (t C ha^-1^)
	Stem (Ws)	Branch (Wb)	Leaf (Wl)	Total	
**DDF**	51.43 ± 19.79	10.34 ± 4.40	1.82 ± 0.62	63.60 ± 27.80	29.89 ± 11.65
**MDF**	56.54 ± 17.60	11.07 ± 3.67	2.06 ± 0.60	69.67 ± 21.83	32.74 ± 10.26
**Average**	52.25 ± 19.20	10.46 ± 4.23	1.86 ± 0.61	64.57 ± 24.02	30.35 ± 11.29

DDF = dry dipterocarp forest, MDF = mixed deciduous forest.

Regarding species contribution to the above-ground biomass in each forest type, *Shorea obtusa* (14 t ha^-1^) had the highest biomass in the DDF, followed by *S*. *siamensis* (9.64 t ha^-1^), *Canarium subulatum* (5.27 t ha^-1^), *Sindora siamensis* (5.14 t ha^-1^), and *Xylia xylocarpa* (4.61 t ha^-1^), respectively. In contrast, *Pterocarpus macrocarpus* (6.27 t ha^-1^) was the species that contributed the most biomass to the MDF, followed by *Lagerstroemia duperreana* (5.86 t ha^-1^), *Garuga pinnata* (5.84 t ha^-1^), *Millettia brandisiana* (4.93 t ha^-1^), and *M*. *leucantha* (4.88 t ha^-1^), respectively.

The forest type cover interpretation of the Landsat images by RF classifier found that the overall accuracy was 91.49% and the Kappa hat coefficient was 85.21%. Application of the CA-Markov model, with a Receiver Operating Characteristic (ROC) curve score of 0.82, predicted changes with a high degree of accuracy.

**Fig 5:**
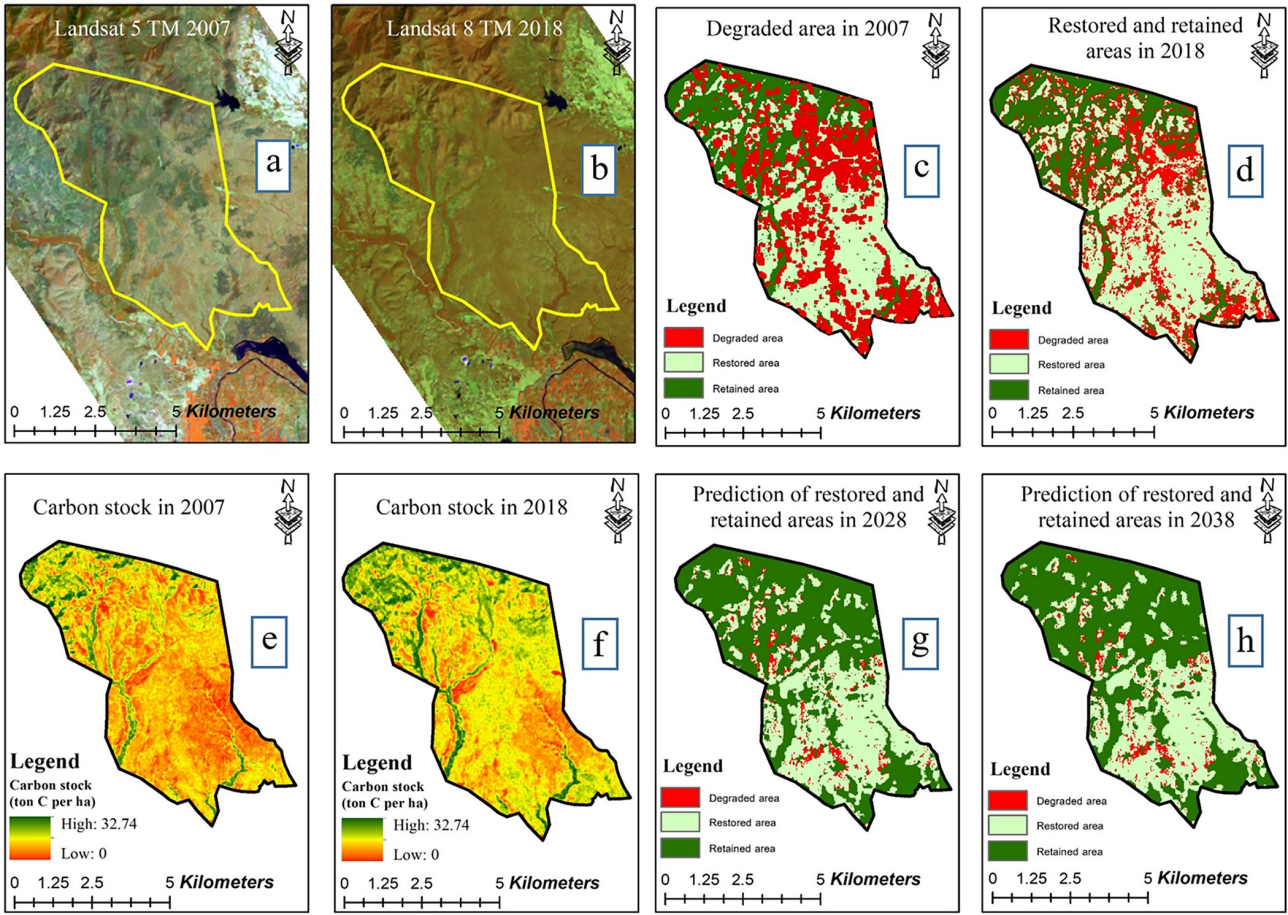
Carbon stock in Ban Mae Chiang Rai Lum Community Forest in various years of CFM implementation. (a, b) Landsat images in 2007 and 2018, (c, d) degraded, restored, and retained areas in 2007 and 2018, (e, f) Carbon stock in 2007 and 2018, and (g, h) Prediction for 2028 and 2038.

As reflected in Table 5, during implementation of CFM, the degraded forest area decreased significantly from 992 ha in 2007 to 143 ha in 2018. In contrast, the restored area dramatically increased from 140 ha in 2007 to 865 ha in 2018. It is projected that the degraded area will diminish further in 2028 and 2038 to 123 ha and 108 ha, respectively, while the restored area will continue to expand to 1,112 ha in 2028 and 1,251 ha in 2038. The projections reflect the most consequential changes in the restored and degraded areas, whereas less significant changes are predicted for the retained area. Though increasing from 2007 to 2018 to an area of 2,917 ha, slight, continual decreases are projected for 2028 (2,690 ha) and 2038 (2,566 ha). The total above-ground biomass throughout the community forest revealed that from 2007 to 2018, carbon stock increased under CFM by 21.82%. Prediction are that carbon stock will increase 24.51% from 2018 to 2028 and an additional 23.06% from 2028 to 2038 (Table 6). Overall, it was estimated that the annual carbon stock in the entire CFM area increased 3,829 t C or 2.89% over the 30-year period under CFM. The changes in forest area and carbon stock between degraded, restored, and retained areas during previous and projected periods of CFM are shown in Fig 5.

**Table 5:** Above-ground biomass and carbon stock by area type in Ban Mae Chiang Rai Lum Community Forest in 2007 & 2018 and projected for 2028, and 2038.

Area type	2007			2018			2028			2038		
	Area (ha)	AGB (t)	CS (t C)	Area (ha)	AGB (t)	CS (t C)	Area (ha)	AGB (t)	CS (t C)	Area (ha)	AGB (t)	CS (t C)
**DA**	992	10,371	4,874	143	5,776	2,715	123	3,200	1,504	108	2,561	1,204
**RA**	140	12,030	5,654	865	22,325	10,493	1,112	73,012	34,316	1,251	99,659	46,840
**ReA**	2,793	259,710	122,064	2,917	315,558	148,312	2,690	351,677	165,288	2,566	388,158	182,434
**Total**	3,925	282,110	132,592	3,925	343,660	161,520	3,925	427,889	201,108	3,925	490,378	247,475

DA = Degraded area, RA = Restored area, ReA = Retained area, AGB = Above-ground biomass, CS = Carbon stock.

**Table 6:** Actual and projected carbon stock change during CFM implementation.

Area type	2007–2018		2018–2028		2028–2038		Average annual rate	
	CS (t C)	% Change	CS (t C)	% Change	CS (t C)	% Change	CS (t C)	% Change
**DA**	−2,159	−44.30	−1,211	−44.60	−300	−19.95	+122	−2.51
**RA**	+4,839	+85.59	+23,823	+227.04	+12,524	+36.50	+1,372	+24.28
**ReA**	+26,248	+21.50	+16,976	+11.45	+17,146	+10.37	+2,012	+1.65
**Total**	+28,928	+21.82	+39,588	+ 24.51	+46,367	+23.06	+3,829	+2.89

DA = Degraded area, RA = Restored area, ReA = Retained area, CS = Carbon stock.

## Discussion

### The deciduous forests’ structure and diversity of species

Ban Mae Chiang Rai Lum Community Forest’s 129 species of 3,769 trees in 43 families collectively exhibit a larger diversity than recorded in other deciduous forests in Thailand [23, 62, 63]. The ecological characteristics of the forests reflected in Table 2 reveal that the mean of the species diversity indices in the community forest (H′ = 2.48) was considered mid-range when compared with other deciduous forests in northern Thailand [64–67]. These results suggest the potential for diverse tree species providing ecosystem services to support rural livelihoods, a finding similar to that in previous studies [68–70].

In both the DDF and MDF, tree density decreased as DBH increased (Fig 4). Represented graphically, this pattern forms an inverted-J shape. This is characteristic of a forest wherein trees regenerate consistently as found in previous studies [71–74]. This is a positive indicator of future natural regeneration of tree species in the forests [9, 75, 76]. The result also revealed the height-class of tree species was normally distributed, resulting in a balanced height class size.

As reflected in Table 3, the DDF was predominantly populated by these tree species: *Shorea obtusa*, *S*. *siamensis*, *Xylia xylocarpa*, *Sindora siamensis*, and *Canarium subulatum*, while *Millettia leucantha*, *Lagerstroemia duperreana*, *Millettia brandisiana*, *Antidesma sootepense*, and *Pterocarpus macrocarpus* were the principal species in the MDF. This is consistent with previous studies of similar forests of this type in Thailand wherein these species were prominent and of significant importance [18, 22, 23, 63, 77, 78].

Our identification of the dominant species in each forest type based on structural features, basal area, and relative dominance, is consistent with previous studies [14, 16]. In addition, we found only a 28.68% similarity in species between the DDF and MDF. Although, our results showed that the difference in density, basal area, and species diversity (H′) were not statistically significant (Table 2), the species composition of the forest structures were nonetheless distinct. This heterogeneity of vegetation patterns between the different forest types could contribute to the difference in the volumes of above-ground biomass and carbon in the Ban Mae Chiang Rai Community Forest.

### The estimation of above-ground biomass and carbon stock

The estimate of the average above-ground biomass of 64.57 t ha^-1^ projected to an average carbon stock of 30.35 t C ha^-1^ in the community forest (Table 4). Similar calculations have been made in other deciduous forests in Thailand [26, 27, 79]. These results highlight the role of deciduous forests in the community forest as carbon sinks that absorb CO_2_ from the atmosphere. However, our carbon stock estimates in this study are lower than those in other areas. This would be related to the massive number of small and young trees populating the community forest.

Several studies have shown that trees with a DBH class size of > 20 cm had greater potential to capture CO_2_ than other size classes [26, 80]. In this study, approximately 80% of the trees in the forest were between 5–20 cm (Fig 4).

Historical encroachment, illegal logging, and deforestation robbed the forest of older and larger trees. Positive regeneration efforts are reflected in the planting of new trees under CFM since 2008. Yet, when compared to other studies, this community forest is composed of an inordinate number of younger and smaller trees with a lower capacity for carbon capture. As such, nurturing younger trees, conserving the existing adult trees and permitting natural regeneration are imperative to reach carbon sequestration potential.

The MDF had a higher rate of carbon sequestration than the DDF. This result is similar to findings in a study of Doi Suthep-Pui National Park, also in northern Thailand, wherein it was determined that the total above-ground biomass of the MDF was higher than in other forest ecosystems, including DDF [9]. Dense canopy cover and a greater numbers of trees are both positively related to carbon sequestration rates [81, 82]. Myo et al. (2016) reported that favorable site conditions related to soil moisture, texture, and organic matter led to more growth stages and numbers of species in the MDF than the DDF.

DDFs in Thailand are generally limited by environmental factors that impact plant growth. DDFs often occur in areas with a mean annual rainfall of 1,000–1,500 mm compared with 1,000–1,800 mm in MDF [16, 17]. MDFs typically grow on moderate fertile loam soil while DDFs have more sandy lateritic soil [16, 17, 19]. Moreover, forest fires are relatively common in deciduous forests during dry season, especially in a DDF [14, 18, 20]. Frequent recurrence of fires in a DDF leads to a loss of major nutrients such as nitrogen, phosphorus, calcium, and potassium, causing a reduction in long-term ecosystem productivity [83]. These features all contribute to and explain the higher carbon stock on the MDF.

Forests can provide different rates of biomass depending on the dominant species in the forest [7]; carbon content in tropical species varied widely from 41.9–51.6% [8]. Increased tree growth can yield more carbon stock underscoring the importance of restoration to promote carbon sequestration [84]. In the DDF, *Shorea obtusa*, *S*. *siamensis*, *Canarium subulatum*, *Sindora siamensis*, and *Xylia xylocarpa* provided the highest biomass. The study of Asanok et al. (2020) demonstrated that DDF species including *Shorea obtusa* and *S*. *siamensis* were strongly and positively affected by photosynthetically active radiation [85]. This suggests that they are pioneer light demander species and are able to survive under extreme drought conditions. In contrast, MDF species such as *Pterocarpus macrocarpus*, *Lagerstroemia duperreana*, *Garuga pinnata*, *Millettia brandisiana*, and *M*. *leucantha* provided the most CO_2_. MDF species are generally found in dense canopies when compared with a DDF that was considered to be more open canopied. This suggests that some species are shade tolerant species in the deciduous forests. Therefore, some tree species may be more suitable for replanting in certain types of forests to accelerate natural succession and maximize the storage of carbon.

### The impact of CFM on reducing carbon emissions

The historical changes in LULC of the Ban Mae Chiang Rai Lum Community Forest revealed in Table 5 and Fig 5 show that total above-ground biomass and carbon stock of the community forest increased from 2007 to 2018 under CFM and were projected to continue to increase from 2018 to 2028 and 2038 under ongoing CFM. Over time, the restored area expanded as the degraded area (and to a lesser extent, the retained area) contracted. This can reflect increasing tree density, growth rate, and the successful regeneration of additional species in the community forest under CFM. It implies effective local management of forest resources and a resulting increase in carbon stock over time. There are numerous possible explanations for this.

Partly in response to encroachment and illegal logging, CFM was implemented in 2008. Collaborative management efforts between the government and local residents resulted in program of forest plantation, fire protection, patrol, and the utilization of check dams. As the result, the community forest was restored, and the damage caused by years of deforestation and degradation was mitigated. It is evident that the community forest sector, through successful management, can play a significant role in global CO_2_ capture and ultimately in reducing the CO_2_ emissions from deforestation and forest degradation. This study in the Ban Mae Chiang Rai Lum community forest, as with similar previous studies, is an example of this potential [28–32].

For the 30 years subsequent to the initiation of CFM in 2008, the projections of carbon stock changes reflect an annual carbon accumulation rate of 3,829 t C. Specifically, from 2007 to 2018, carbon stock increased a total of 28,928 t C, while it was projected to increase during 2018–2028 and 2028–2038 to 39,588 t C and 46,367 t C, respectively. Overall, annual change in carbon stock under CFM implementation from 2007 and projected through 2038 was 0.98 t C h^-1^ or an annual increase of 2.89% (Table 6). These estimates of future carbon stock varied with those of other studies. In deciduous forests of Korea, Lee et al. (2018) found that carbon stocks increased 15.55% between 2010 and 2015 (approximately 3.11% annually) [86]. Bhat and Ravindranath (2011) reported that accumulation rates of carbon ranged from 0.31–3.19 t C h^-1^ per year in tropical rainforests of India [87]. In the community forests of Dolakha, Nepal, the annual rate of carbon sequestration was 2.19 t C h^-1^ [30].

As the volume of trees incrementally expands during each growing season, it is expected that the rate of increase of carbon stock would also increase over 10, 20, and 30-year periods of time. In the current study, however, the estimated amount of carbon increased 21.82% between 2007 and 2018 and was projected to increase by an additional 24.51% through 2028, and by 23.06% through 2038. From 2028 through 2038, the rate of projected increase actually slowed by 1.45%. A possible explanation for this result may be related to the level of effectiveness of forest management.

In a study conducted in the same area, Thammanu et al. (2021) found that distance from communities was negatively related to the composition and distribution of trees in the community forest [88]. This supports a conclusion that that utilization of forest resources affects tree species. In addition, dipterocarp species were often used as firewood in households, and since dipterocarp species are crucial carbon stock contributors, this could inform the impact of CFM on carbon stock in the community forest.

Previous studies have demonstrated that utilization of forest resources was closely associated with tree species. Extraction of forest resources can alter species composition and distribution [89] and unsustainable utilization of forest resources can result in decreased regeneration of tree species and tree populations [90, 91]. Over-exploitation of forest resources not only affects species diversity, but also has a long term, harmful impact on ecosystem health and resiliency [92]. Consequently, using resources in an inappropriate manner, or over-use of resources, may decrease the forest productivity that provide benefits to communities. Thus, continued improper utilization of forest resources could theoretically inhibit the continued increase of carbon stock in the years to come.

Myriad factors contribute to forest fires in Thailand. Primary among them is NTFP harvesting in forest areas [93]. People in the remote area rely on their indigenous knowledge for collection of NTFPs such as edible plants, bamboo shoots, ant eggs, small animals, honey bee, mushrooms *[94, 95]* and fires are often used as a management tool for harvesting NTFPs [96]. This human activity combined with the 5–6 months of dry season and drought conditions of deciduous forests [18, 20] make these areas sensitive to regular forest fires and the harmful impact they have on carbon stock. Tree species in deciduous forests are naturally able to adapt to grow in drought areas with poor soil properties, high aridity, and fire disturbance [18, 97, 98], but long-term extreme conditions can still have a negative impact on tree species regeneration, productivity and on the forest ecosystem [22, 83].

However, another reason would be forests tend to stabilize their growth as they reach maturity over time. In general, early stages of stand development feature higher growth efficiencies, and older growth forests can have ecosystem net productivity near zero and even possibly negative [99, 100]. As such, trees may reach maximum levels in an old-growth stage resulting in a slower rate of carbon stock increases. Understanding the characteristics and dynamics of deciduous forests and human behavior helps to inform a strategy to manage forests and limit the impact of that behavior.

### The opportunities for REDD+ in the community forest

As reflected in Table 5, in 2018 the total carbon stock in Ban Mae Chiang Rai Lum Community Forest’s complete 3,925 ha area was estimated to be 132,592 t C or 486,612.64 tCO_2_e by applying the standard conversion rate of 1 ton C / 3.67 tons of CO_2_e [101]. Comparing carbon stock in pre-CFM 2007 with that in 2018 after a period of CFM reflects an increase of 28,928 t C or 106,165.76 tCO_2_e (Table 6).

A carbon offset price depends on several factors. Not knowing project costs, buyer preferences and other factors that contribute to and affect pricing limits the ability to determine an actual price or identify overall economic benefit to a community and increases uncertainty in speculating about such benefits.

The average price for Forestry and Land Use activities was used to derive an estimate of the carbon offset in this study [102]. Assuming a carbon offset price in the voluntary market of US $3.20 per ton CO_2_e in 2018, this carbon stock would have returned US $339,730.43 or US $86.56 per ha to the communities. By comparison, other studies around the world have generated carbon savings estimates. Under CFM in a dry forest in West Africa, it was estimated to be US $120 per ha [28]. In Nepal, the benefits of REDD+ to forest communities was US $152 per ha in the Ludikhola watershed and US $29 per ha in the Kayarkhola watershed [32].

Gurung et al. (2015) suggested that protection of the community forest through effective forest mechanisms, more so than in government managed forests, could result in higher density of carbon [31]. As such, our findings indicate that increased carbon sequestration under CFM could be an attractive opportunity to provide subsistence and other economic benefits to rural communities similar to other countries.

Currently, there are over 17,400 villages in Thailand [36]. Ban Mae Chiang Rai Lum Community Forest is an example of collaborative success managing forest resources between local people and the government, success that can have positive and significant environmental and economic benefits.

National and widespread efforts to involve community forests in the expansive policies and strategies of REDD+ and its international carbon market would be progress for local communities toward sustainable forest management. Focused efforts to protect forest resources can contribute to addressing the problem of climate change through the reduction of CO_2_ emissions as well as provide significant economic benefits.

Historically, a lack of a specific framework for implementing CFM inhibited the effectiveness of local management in developing strategies and providing technical knowledge and assistance to CFM members [95]. To an extent, this was addressed by Community Forest Act B.E. 2562 wherein local decision making authority was formally provided thereby incentivizing participation, expanding local control and creating new opportunities to benefit from successful management.

## Conclusions

Our study provides insights into the structure of two deciduous forests. Although forest structure and species composition varied between the DDF and MDF, there was no difference in above-ground carbon stock. Overall, the potential for carbon-market based economic value inuring to the community indicates that REDD+ and its policies and strategies could be a good opportunity under CFM. Resulting benefits to the environment as well as financial benefits to the community could incentivize more involvement in managing forest resources in other areas. Rapidly increasing CFM projects in Thailand and laws permitting more local decision making can also facilitate implementation.

However, it is projected that the rate of carbon stock increase in the community forest will slow over time. This suggests that human activity and unstainable forest management may negatively impact the accumulation of carbon stock. Consequently, the management practices of Ban Mae Chiang Rai Lum should address utilization of forest resources. Promoting NTFP utilization sustainably should be prioritized as a strategy crucial to maintaining carbon stock in the community forest. Forest fire protection measures and restoration policies to reduce the impact of drought conditions as exacerbated by human activity are important components of effective forest management, all of which facilitate tree species abundance and proliferation while reducing carbon emissions.

Therefore, community forest projects implementing REDD+ in Thailand could be a crucial component and strategy toward successfully achieving the country’s target of reducing its greenhouse gas emissions by 20% by 2030. Nevertheless, more encompassing data regarding structure and carbon sequestration in community forests nationwide is not presently available. Taking advantage of REDD+ opportunities and realizing all potential benefits, economic and otherwise, requires more extended studies of community forests in Thailand. Only through such an assessment can the local people and governments be fully prepared for REDD+ implementation.

## Supporting information

S1 Data (XLSX)

## References

[1] Brown S, Lugo AE. The storage and production of organic matter in tropical forests and their role in the global carbon cycle. Biotropica. 1982; 14: 161–187.

[2] Bonan GB. Forests and climate change: Forcings, feedbacks, and the climate benefits of forests. Science. 2008; 320: 1444–1449. doi: 10.1126/science.1155121 18556546

[3] Pan Y, Birdsey RA, Fang J, Houghton R, Kauppi PE, Werner A, et al. A large and persistent carbon sink in the world’s forests. Science. 2011; 333: 988–993. doi: 10.1126/science.1201609 21764754

[4] Santillin M, Moutinho P, Schwartzman S, Nepstad D, Curran L, Nobre C. Tropical deforestation and the Kyoto protocol. Clim. Change. 2005; 71: 267–276. doi: 10.1007/s10584-005-8074-6

[5] Gibbs HK, Brown S, Niles JO, Foley JA. Monitoring and estimating tropical forest carbon stocks: making REDD a reality. Environ. Res. Lett. 2007. doi: 10.1088/1748-9326/2/4/045023

[6] ONEP. Second biennial update report of Thailand. ONEP; 2018.

[7] Kaewkrom P, Kaewkla N, Thummikkapong S, Punsang S. Evaluation of carbon storage in soil and plant biomass of primary and secondary mixed deciduous forests in the lower northern part of Thailand. Afri. J. Environ. Sci. Technol. 2011; 5: 8–14.

[8] Thomas SC, Martin AR. Carbon content of tree tissues: A synthesis. Forests. 2012; 3: 332–352. doi: 10.3390/f3020332

[9] Hermhuk S, Chaiyes A, Thinkampheang S, Danrad N, Marod D. Land use and above-ground changes in a mountain ecosystem, Northern Thailand. J. For. Res. 2019; 31: 1733–1742. doi: 10.1007/s11676-019-00924-x

[10] IPCC. 2006 IPCC guidelines for national greenhouse gas inventories. IGES: Japan; 2006.

[11] RFD. Executive summary. RFD; 2019.

[12] Smitinand T. The distribution of the dipterocarpaceae in Thailand. In: Proceedings of The 11th Pacific Science Congress, Tokyo, Japan, 1966 Sept 1. pp. 67–77.

[13] Smitinand T. Vegetation and ground cover of Thailand. Department of Forest Biology: Kasetsart University; 1977.

[14] Kutintara U. Structure of the dry dipterocarp forest. Ph.D. Thesis, Colorado State University, Colorado. 1975.

[15] FAO. Thailand forestry outlook study. FAO; 2009.

[16] Bunyavejchewin S. Canopy structure of the dry dipterocarp forest of Thailand. In: Thai Forest Bulletin, RFD; 1983. pp. 1–93.

[17] Santisuk T. An account of the vegetation of Northern Thailand; Franz Steiner Verlag Wiesbaden; 1988.

[18] Bunyavejchewin S, Baker PJ, Davies SJ. Seasonally dry tropical forests in continental Southeast Asia-Structure, composition, and dynamics. In: The ecology and conservation of seasonally dry forests in Asia, McShea W, Davis S, Bhumpakphan N, editors. Smithsonian Institution Scholarly Press; 2011. pp. 9–35.

[19] Bunyavejchewin S. Analysis of the tropical dry deciduous forest of Thailand. II. Vegetation in relation to topographic and soil gradients. Nat. Hist. Bull. Siam. Soc. 1985; 33: 3–20.

[20] Sukwong S, Dhamanittakul P. Fire ecology investigations in dry dipterocarp forest. In: Proceedings of the National Forestry Conference, Bangkok, Thailand; 1977. pp. 41–56.

[21] Khemnark C. Natural regeneration of the deciduous forests in Thailand. Kasetsart University; 1979. pp. 31–43.

[22] Marod D, Kutintara U. Yarwudhi C, Tanaka H, Nakashisuka T. Structural dynamics of a natural mixed deciduous forest in Western Thailand. J. Veg. Sci. 1999; 10: 777–786.

[23] Kabir ME, Webb EL. Saving a forest: The composition and structure of a deciduous forest under community management in Northeast Thailand. Nat. Hist. Bull. Siam. Soc. 2006; 54: 239–260.

[24] Larpkerna P, Eriksen MH, Waiboonya P. Diversity and uses of tree species in the deciduous dipterocarp forest, Mae Chaem District, Chiang Mai Province, Northern Thailand. NUTST. 2017; 3: 43–55.

[25] Mianmit N, Jintana V, Sunthornhao P, Kanhasin P, Takeda S. Contribution of NTFPs to local livelihood: A case study of Nong Sai Sub-district of Nang Rong District under Buriram Province in Northeast Thailand. J. Agro. Environ. 2017; 11: 123–128.

[26] Terakunpisut J, Gajaseni N, Ruankawe N. Carbon sequestration potential in aboveground biomass of Thong Pha Phum National Forest Thailand. App. Ecol. Environ. Res. 2007; 5: 93–102.

[27] Chaiyo U, Garivait S, Wanthongchai K. Carbon storage in above-ground biomass of tropical deciduous forest in Ratchaburi Province, Thailand. World Acad. Sco. Eng. Technol. 2011; 58: 636–641. doi: 10.13140/2.1.3011.0723

[28] Skutsch MM, Ba L. Crediting carbon in dry forests: The potential for community forest management in West Africa. For. Policy Econ. 2009; 12: 264–270. doi: 10.1016/j.forpol.2009.12.003

[29] Pandey SS, Maraseni TN, Cockfield G. Carbon stock dynamics in different vegetation dominated community forests under REDD+: A case from Nepal. For. Ecol. Manag. 2014; 327: 40–47. doi: 10.1016/j.foreco.2014.04.028

[30] Shrestha S, Karky BS, Karki S. Case study report: REDD+ pilot project in community forests in three watersheds of Nepal. Forests. 2014; 5: 2425–2439. doi: 10.3390/f5102425

[31] Gurung MB, Bigsby H, Cullen R, Manandhar U. Estimation of carbon stock under different management regimes of tropical forest in the Terai Arc Landscape, Nepal. For. Ecol. Manag. 2015; 356: 144–152. doi: 10.1016/j.foreco.2015.07.024

[32] Pandit R, Neupane PR, Wagle BH. Economics of carbon sequestration in community forests: Evidence from REDD+ piloting in Nepal. J. For. Econ. 2017; 26: 9–29. doi: 10.1016/j.jfe.2016.11.002

[33] Pagdee A, Kim YS, Daugherty PJ. What makes community forest management successful: A meta-study from community forests throughout the world. Soc. Nat. Resour. 2007; 19: 33–52. doi: 10.1080/08941920500323260

[34] RFD. Implementation guidelines for community forest projects of the Royal Forest Department: RED; 2014. (In Thai)

[35] Royal Thai Government. Community forest act B.E. 2562. Royal Thai Government; 2019. pp. 71–103.

[36] RFD. Community forest project approval between 2000 –present. 2017 Feb 2 [cited 2020 Jan 30]. Availabe from: http://www.forest.go.th/community-extension/2017/02/02/

[37] Thai Meteorological Department. Daily meteorology. Northern Meteorological Center; 2018.

[38] Thammanu S, Han H, Marod D, Zang L, Jung Y, Soe KT, et al. Non-timber forest product utilization under community forest. Forest Sci. Technol. 2021; 17: 1–15. doi: 10.1080/21580103.2020.1862712

[39] Lise W. Factors influencing people’s participation in forest management in India. Ecol. Econ. 2000; 34: 379–392. doi: 10.1016/S0921-8009(00)00182-8.

[40] Jumbe CBL, Angelsen A. Forest dependence and participation in CPR management: Empirical evidence from forest co-management in Malawi. Ecol. Econ. 2007; 62: 661–672. doi: 10.1016/j.ecolecon.2006.08.008

[41] Coulibaly-Lingani P, Savadogo P, Tigabu M, Oden P-C. Factors influencing people’s participation in the forest management program in Burkina Faso, West Africa. For. Policy Econ. 2011; 13: 292–302. doi: 10.1016/j.forpol.2011.02.005

[42] Tugume P, Buyinza M, Namaalwa J, Kakudidi EK, Mucunguzin P, Kalema J, et al. Socio-economic predictors of dependence on non-timber forest products: Lessons from Mabira Central Forest Reserve Communities. J. Environ. Agric. Sci. 2015; 4: 195–214. doi: 10.15640/jaes.v4n2a23

[43] Soe KT, Youn Y. Perceptions of forest-dependent communities toward participation in forest conservation: A case study in Bago Yoma, South-Central Myanmar. For. Policy Econ. 2019; 100: 129–141. doi: 10.1016/j.forpol.2018.11.009

[44] RFD. Study of carbon sequestration and biodiversity in the Ban Mae Chiang Rai Community Forest, Northern Thailand. RFD; 2017. (In Thai)

[45] Avery TE, Burkhart HE. Forest measurements. McGraw-Hill Publishing Company; 1983.

[46] Asrat Z, Tesfaye Y. Training manual on: Forest inventory and management in the context of SFM and REDD+. Hawassa University; 2013.

[47] ANSAB. Participatory inventory of non-timber forest products. ANSAB; 2010.

[48] UNFCCC. Measurements for estimation of carbon stocks in afforestation and reforestation project activities under the clean development mechanism: A field manual. UNFCCC; 2015.

[49] Abino AC, Castillo JAA, Lee YJ. Assessment of species diversity, biomass and carbon sequestration potential of a natural mangrove stand in Samar, the Philippines. Forest Sci. Technol. 2013; 10: 2–8. doi: 10.1080/21580103.2013.814593

[50] McCune B, Grace J. Analysis of ecological communities. MjM Software Design; 2002.

[51] Magurran AE. Measuring biological diversity. Blackwell Publishing; 2004.

[52] Magurran AE. Ecological diversity and its measurement. Groom Helm Ltd; 1998.

[53] Curtis JT, McIntosh RP. An upland forest continuum in the Prairie‐Forest Border Region of Wisconsin. Ecology. 1951; 32: 476–496. doi: 10.2307/1931725

[54] Ogawa H, Yod K, Ogino K, Kira T. Comparative ecological studies on three main types of forest vegetation in Thailand. II. Plant Biomass. Nature and life in Southeast Asia. 1965; 4: 49–80.

[55] Dupuis C, Lejeune P, Michez A, Fayolle A. How can remote sensing help monitor tropical moist forest degradation?-A systematic review. Remote Sens. 2020; 12: 1087. doi: 10.3390/rs12071087

[56] Srichaichana J, Trisurat Y, Ongsomwang S. Land use and land cover scenarios for optimum water yield and sediment retention ecosystem services in Klong U-Tapao Watershed, Songkhla, Thailand. Sustainability. 2019; 11: 2895. doi: 10.3390/su11102895

[57] Mondal MS, Sharma N, Garg PK, Kappas M. Statistical independence test and validation of CA Markov land use land cover (LULC) prediction results. Egypt. J. Remote. Sens. Space Sci. 2016; 19: 259–272. doi: 10.1016/j.ejrs.2016.08.001

[58] Hamad R, Balzter H, Kolo K. Predicting land use/land cover changes using a CA-Markov model under two different scenarios. Sustainability. 2018; 10: 3421. doi: 10.3390/su10103421

[59] Mannan A, Liu J, Zhongke F, Khan TU, Saeed S, Mukete B, et al. Application of land-use/land cover changes in monitoring and projecting forest biomass carbon loss in Pakistan. Glob. Ecol. Conserv. 2019; 17: e00535. doi: 10.1016/j.gecco.2019.e00535

[60] McCune B, Mefford M.J. PC-ORD: Multivariate analysis of ecological data. MjM Software Design: Gleneden Beach; 2006.

[61] R Development Core Team. R: A language and environment for statistical computing. 2019 Dec 12 [cited 2020 Nov 20]. Available from: https://www.r-project.org/

[62] Larpkern P, Moe SR, Totland Ø. The effects of environmental variables and human disturbance on woody species richness and diversity in a bamboo–deciduous forest in northeastern Thailand. Ecol. Res. 2009; 24: 147–156. doi: 10.1007/s11284-008-0492-2

[63] Lamotte S, Gajaseni J, Malaisse F. Structure diversity in three forest types of north-eastern Thailand (Sakaerat Reserve, Pak Tong Chai). Biotechnol. Agron. Soc. and Environ. 1998; 2: 192–202.

[64] DNP. A permanent plot sampling project in a dry dipterocarp forest in Mae Ping National Park: Chiang Mai, Lum Phun and Tak Provinces. DNP; 2015. (In Thai)

[65] DNP. The biological diversity in protected area: Chiangdao Wildlife Santuary. Protected Area Regional Office 16; 2016. (In Thai)

[66] Papakjan N, Asanok L, Thapyai C. Plant community and environment factors influence on the natural regeneration on tree in the forest edge of deciduous dipterocarp forest and mixed deciduous forest after highland maize cropping at Mae Khum Mee Watershed, Phrae Province. In: Proceedings of The 6th of Thai Forest Ecological Research Network, Mahidol University, Nakhon Pathom, Thailand, 2017 Dec 19–20. pp. 123–131. (In Thai)

[67] Pothawong N, Sringernyuang K, Seetakoses P, Fongmoon S, Kumyong S. Forest structure, diversity and utilization under the community resource management of the community forest at Baan Ta Pa Pao, Thapladuk Sub-District, Lamphun Province. In: Proceedings of The 5th of Thai Forest Ecological Research Network, Kasetsart University, Bangkok, Thailand, 2015 Dec 16–17. pp. 56–65. (In Thai)

[68] Kim S, Sasaki N, Koike M. Assessment of non-timber forest products in Phnom Kok community forest, Cambodia. Asia Eur. J. 2008; 6: 345–354. doi: 10.1007/s10308-008-0180-4

[69] Kumar V. Impact of non timber forest produces (NTFPs) on food and livelihood security: An economic study of tribal economy in Dang’s District of Gujarat, India. Int. J. Agric. Environ Biotechnol. 2015; 8: 387–404. doi: 10.5958/2230-732X.2015.00047.9

[70] Rijal S, Adhikari S, Pant RR. Non-timber forest products and livelihood linkages: A case of Lamabagar, Nepal. World Acad. Sci. Eng. Technol. 2019; 13: 326–331.

[71] Culmsee H, Leuschner C, Moser G, Pitopang R, Silman M. Forest aboveground biomass along an elevational transect in Sulawesi, Indonesia, and the role of Fagaceae in tropical montane rain forests. J. Biogeogr. 2010; 37: 960–974. doi: 10.1111/j.1365-2699.2009.02269.x

[72] Alvarez E, Duque A, Saldarriaga J, Cabrera K, Salas GL, Valle I, et al. Tree above-ground biomass allometries for carbon stocks estimation in the natural forests of Colombia. For. Ecol. Manag. 2012; 267: 297–308. doi: 10.1016/j.foreco.2011.12.013

[73] Magalhães TM, Seifert T. Tree component biomass expansion factors and root-to-shoot ratio of Lebombo ironwood: Measurement uncertainty. Carbon Balance Manag. 2015; 10: 9. doi: 10.1186/s13021-015-0019-4 26316881 PMC4544549

[74] Zhao L, Xiang W, Li J, Lei P, Deng X, Fang X, et al. Effects of topographic and soil factors on woody species assembly in a Chinese Subtropical Evergreen Broadleaved Forest. Forests. 2015; 6: 650–669. doi: 10.3390/f6030650

[75] Kimmins JP. Forest ecology. Macmillan Publishing Company; 1987.

[76] Myo KK, Thwin S, Khaing N. Floristic composition, structure and soil properties of mixed deciduous forest and deciduous dipterocarp forest: Case study in Madan Watershed, Myanmar. Am. J. Plant Sci. 2016; 7: 279–287. doi: 10.4236/ajps.2016.72027

[77] Teejuntuk S, Sahunalu P, Sakura K, Sungpalee W. Forest structure and tree species diversity along an altitudinal gradient in Doi Inthanon National Park, Northern Thailand. Tropics. 2003; 12: 85–102. doi: 10.3759/tropics.12.85

[78] Khamyong N, Wangpakapattanawong P, Chairuangsri S, Inta A, Tiansawat P. Tree species composition and height-diameter allometry of three forest types in Northern Thailand. Chiang Mai Univ. J. Nat. Sci. 2018; 17: 289–306. doi: 10.12982/CMUJNS.2018.0021

[79] Bridhikitti A. Soil and biomass carbon stocks in forest and agricultural lands in tropical climates. Songklanakarin J. Sci. Technol. 2017; 39: 697–707.

[80] Pibumrung P, Gajaseni N, Popan A. Profiles of carbon stocks in forest, reforestation and agricultural land, Northern Thailand. J. For. Res. 2008; 19: 11–18. doi: 10.1007/s11676-008-0002-y

[81] Pandey SS, Cockfield G, Maraseni TN. Dynamics of carbon and biodiversity under REDD+ regime: A case from Nepal. Environ. Sci. Policy. 2014; 38: 272–281. doi: 10.1016/j.envsci.2014.01.005

[82] Jithila PJ, Prasadan PK. Carbon storage and sequestration by trees—A study in Western Ghats Wayanad Region. Indian J. Ecol. 2018; 45: 000–000.

[83] Wanthongchai K, Bauhus J, Goldammer JG. Nutrient losses through prescribed burning of aboveground litter and understorey in dry dipterocarp forests of different fire history. Catena. 2008: 74. doi: 10.1016/j.catena.2008.01.003

[84] Kebede B, Soromessa T. Allometric equations for aboveground biomass estimation of Olea europaea L. subsp. cuspidata in Mana Angetu Forest. Ecosyst. Health Sustain. 2018; 4: 1–12. doi: 10.1080/20964129.2018.1433951

[85] Asanok L, Taweesuk R, Papakjan N. Woody species colonization along edge-interior gradients of deciduous forest remnants in the Mae Khum Mee Watershed, Northern Thailand. Int. J. For. Res. 2020. doi: 10.1155/2020/5867376

[86] Lee SJ, Yim JS, Son YM, Son Y, Kim R. Estimation of carbon stocks for national greenhouse gas inventory reporting in South Korea. Forests. 2018; 9: 625. doi: 10.3390/f9100625

[87] Bhat DM, Ravindranath NH. Above–ground standing biomass and carbon stock dynamics under a varied degree of anthropogenic pressure in tropical rain forests of Uttara Kannada District, Western Ghats, India. Taiwania. 2011; 56: 85–96. doi: 10.6165/tai.2011.56(2).85

[88] Thammanu S, Marod D, Han H, Bhusal N, Asanok L, Ketdee P, et al. The influence of environmental factors on species composition and distribution in a community forest in Northern Thailand. J. For. Res. 2021; 32: 649–662. doi: 10.1007/s11676-020-01239-y

[89] Thapa S, Chapman DS. Impacts of resource extraction on forest struture and diversity in Bardia National Park, Nepal. For. Ecol. Manag. 2010; 259: 641–649. doi: 10.1016/j.foreco.2009.11.023

[90] Murali KS, Shankar U, Shaanker RU, Ganeshaiah KN, Bawa KS. Extraction of non-timber forest products in the forests of Biligiri Rangan Hills, India. 2. Impact of NTFP extraction on regeneration, population structure, and species composition. Econ. Bot. 1996; 50: 270–279.

[91] Popradit A, Srisatit T, Kiratiprayoon S, Yoshimura J, Ishida A, Shiyomi M, et al. Anthropogenic effects on a tropical forest according to the distance from human settlements. Sci. Rep. 2015; 14689. doi: 10.1038/srep14689 26434950 PMC5155698

[92] Rowe RJ. Environmental and geometric drivers of small mammal diversity along elevational gradients in Utah. Ecography. 2009; 32: 411–422. doi: 10.1111/j.1600-0587.2008.05538.x

[93] RFD. Causes of forest fires. [cited 2021 Jan 19]. Availabe from: https://www.forest.go.th

[94] Delang CO. Indigenous systems of forest classification: Understanding land use patterns and the role of NTFPs in shifting cultivators’ subsistence economies. Environ. Manage. 2006; 37: 470–486. doi: 10.1007/s00267-005-0097-2 16465561

[95] Salam MA, Noguchi T, Pothitan R. Community forest management in Thailand: Current situation and dynamics in the context of sustainable development. New Forest. 2006; 31: 273–291. doi: 10.1007/s11056-005-7483-8

[96] Makarabhirom P, Ganz D, Onprom S. Community involvement in fire management: Cases and recommendations for community-based fire management in Thailand. In: Proceedings of The International Conference on Community Involvement in Fire Management, Bangkok, Thailand; 2002. pp. 10–15.

[97] Rundel PW, Boonprakob K. Forest ecosystems of Thailand. In; Seasonal dry tropical forests, Bullock SH, Mooney H, Medina E, editors. Cambridge University Press; 1995. pp. 93–123. doi: 10.1007/BF00329426

[98] Marod D, Kutintara U, Tanaka H, Nakashizuka T. The effects of drought and fire on seed and seedling dynamics in a tropical seasonal forest in Thailand. Plant Ecol. 2002; 161: 41–57. doi: 10.1023/A:1020372401313 11099962

[99] Waring RH. Estimating forest growth and efficiency in relation to canopy leaf area. Adv. Ecol. Res. 1983; 13: 327–354.

[100] Kaufmann MR, Moir WH, Covington WW. Old-growth forests: What do we know about their ecology and management in the southwestern and Rocky Mountain regions. In; Proceedings of Old-Growth Forests in the Southwest and Rocky Mountain Regions, Arizona, USA, 1992 Mar 9–13. pp. 1–11.

[101] Carbon Fix Standard Version 3.0. Criteria and methodology. [cited 2021 Jul 5]. Available from: https://www.co2-sachverstaendiger.de/pdf/CFS%20v30%20Criteria_Methodology.pdf

[102] Donofrio S, Maguire P, Merry W, Zwick S. Forest Trends’ Ecosystem Marketplace. Financing emissions reductions for the future: State of the Voluntary Carbon Markets; 2019.

